# The Paper Mache Bra: A Postsurgical Breast Taping Technique

**DOI:** 10.1097/GOX.0000000000002407

**Published:** 2019-09-30

**Authors:** Amiram Borenstein, Ron Azaria, Roy Inbar, Or Friedman

**Affiliations:** From the * Borenstein Clinic, Tel Aviv, Israel; † Azaria Inbar Private Clinic, Tel Aviv, Israel; ‡ Sackler Faculty of Medicine, Tel Aviv University, Tel Aviv, Israel.

## Abstract

Little has been published about dressing the breast after surgery and the potential benefits of added support to the routine use of a nonwired bra postoperatively. We report a postsurgical breast taping method and suggest its use might help reduce minor postsurgical complications and subsequent impaired scarring.

## INTRODUCTION

Breast procedures are the most common procedures in plastic surgery. The 2018 ASPS statistics quote 313,735 breast augmentation, 29,236 implant removals, 109,638 breast lifts, and 43,591 breast reductions in the United States.^1^ Scars are an unavoidable part of any surgery and primary cause of concern for patients seeking aesthetic breast surgery.^2^ Intrinsic patient-related factors include skin quality, personal scarring tendencies, and preexisting nutritional or medical wound healing problem.^3^ Extrinsic surgeon-related factors include incision planning, atraumatic tissue handling, layered closure for tension distribution, respecting the dermal blood supply, and good epidermal opposition with minimal tension.^3^ Surgeons have very little control over patient-related factors; therefore, an open and honest discussion regarding scarring is critical in any patient evaluation and presurgical planning. Extrinsic factors can be influenced during the surgery itself and complemented by postsurgical care.

Surgeons often use surgical tape to cover their incisions.^4^ We describe our preferred postsurgical breast taping method after breast surgery.

### Breast Taping Technique

Breast taping technique is shown in video (See Video 1 [online], which displays the breast taping technique).

**Video 1.** This video displays the breast taping technique.

#### Clean

The freshly sutured breast was gently wiped, using damp, warm lap pads followed by dry lap pads. Several passes are done to remove blood, serum, and fatty debris. The skin surface should not be wet or shiny at the end.

### Vertical Scar Pattern: First

#### Vertical Scar Taping

A horizontal strip of sterile porous tape (3M, Steri-Strips, St. Paul, MN) was placed over the vertical suture line and around the nipple–areola complex.

### Thick Horizontal: Second

#### Horizontal Taping

The second layer of wide, porous tape (3M Micropore) was placed for spanning the breast. Starting from the lowest part of the vertical scar, defining the IMF. This step usually consists of several horizontal strips with 50% overlap above and below the NAC.

### V-Shape Taping: Third

#### Superomedial to Inferior–Lateral

Next, 4 wide, porous tape strips were placed diagonally over the breast.

#### Superolateral to Inferior–Medial

Similarly, 4 wide, porous tape strips were placed diagonally over the breast.

### Diamond Pattern: Fourth

#### Medial and Lateral Pole Taping

Here, we treat the medial and lateral poles of the breast like the IMF, placing Vertical strips with 50% overlap, defining the convexity of the breast and slightly suspending it using the tape.

### Follow-up

The patients are discharged with the “paper mache bra” and an unwired bra. Patients are seen on POD 1 when the NAC is inspected, and the rest of the taped breast is inspected for discharge or loosening of the tape. Patients can shower with the “paper mache bra” from POD 3. The patients are inspected weekly until POD 21 when the whole “paper mache bra” is easily removed en bloc with little to no discomfort to the patient. (See Video 2 [online], which displays the removal of the “paper mache bra” on POD 21. Note that the “bra” is removed “en bloc” with little to no discomfort to the patient.)

**Video 2.** This Video displays the removal of the “paper mache bra” on POD 21. Note that the “bra” is removed “en bloc” with little to no discomfort to the patient.

## DISCUSSION

The breast is unique in terms of its composition and support structure. The ideal western breast is large and projected, composed mainly of soft glandular tissue and fat. The breast is supported by a series of ligaments that tether the glandular tissue and surrounding skin envelope to the chest wall. These ligaments are inevitably disrupted during breast surgery.^5,6^ We suggest that support of the breast postoperative should be a vital element of any breast procedure—similar to a skin graft “tie over” or an extremity reduction and fixation.

The main advantages of the technique proposed are the custom-made support of the freshly reshaped breast, the ability to shower over it, and permits monitoring of the NAC.

The limitations of this method are that it somewhat lengthens the procedure, and some might have concerns regarding skin colonization and surgical site infection. Regarding the added time, carefully cleaning and taping a single breast takes 3–5 minute and can be done by an assistant. It has been shown by multiple levels 2 and 1 studies that breast dressing up to 6 days indeed increases measured skin colonization, but with no increase of surgical site infection.^7,8^ Moreover, patients tend to comply with postsurgical dressing and even seem to prefer it over a daily exchange.

We strongly feel that this protocol aids in lowering our minor dehiscence complication rates and perhaps even contributes to better scars.
